# The effects of 4 weeks of sport-specific repeated sprint training on upper-body strength quality, anaerobic capacity and punching ability of elite male boxers

**DOI:** 10.3389/fspor.2026.1839505

**Published:** 2026-06-23

**Authors:** Tan Tian, Yongjie Niu, Shanjun Bao

**Affiliations:** 1 School of Sports Training, Wuhan Sports University, Wuhan, China; 2 School of Physical Education, Ningde Normal University, Ningde, China

**Keywords:** anaerobic capacity, boxers, high-intensity interval training, repeated sprint training, sport-specific, strength quality

## Abstract

**Background:**

Repeated sprint training (RST) effectively enhances lower-body performance, yet its effects on upper-body strength and sport-specific punching ability in boxers remain poorly understood.

**Purpose:**

To investigate the effects of a 4-week boxing-specific repeated sprint training intervention on upper-body strength, anaerobic capacity, and punching ability in elite male boxers.

**Methods:**

Sixteen elite male boxers were randomly assigned to an experimental group (EG, *n* = 8) or a control group (CG, *n* = 8). The EG performed three weekly sessions of all-out, sport-specific RST (14 sets of 3 s maximal heavy-bag punches, 10 s rest between sets, 1 min rest between rounds) in addition to conventional training, while the CG continued conventional training only. Upper-body strength (bench press mean propulsive power, MPP), anaerobic capacity (upper-body Wingate peak and mean power), and punching ability (peak punch velocity, total punches in 30 s, mean punch velocity) were assessed before and after the 4-week intervention.

**Results:**

The EG showed large within-group improvements in Wingate peak power (*d* = 1.10, +16.7%) and mean power (*d* = 1.14, +20.6%), with large between-group effect sizes post-intervention (peak power: *d* = 1.60; mean power: *d* = 1.61). Bench press MPP increased substantially within the EG (*d* = 1.15, +20.4%), and although the between-group difference did not reach statistical significance (*p* = 0.059), the effect size was large (*d* = 1.03). The EG also exhibited large within-group improvements in peak punch velocity (*d* = 1.13, +7.3%), total punches (*d* = 1.15, +16.8%), and mean punch velocity (*d* = 1.35, +14.9%). The between-group difference in total punches was not significant (*p* = 0.074) but corresponded to a large effect size (*d* = 0.97).

**Conclusion:**

Incorporating three weekly sessions of boxing-specific RST into conventional training over 4 weeks significantly improved upper-body anaerobic capacity and produced practically meaningful enhancements in explosive strength and punching endurance in elite male boxers.

## 1 Introduction

Repeated-sprint training (RST) is an effective training method for improving various physical performance metrics in athletes, including strength, endurance, and aerobic capacity. It involves performing multiple sets of all-out efforts lasting 3–10 s, with recovery intervals at either 45% of the final speed from a submaximal fitness test or 60% of the final speed from a progressive load test. Recovery durations range from 15 to 60 s. However, research on RST is less extensive than research on sprint interval training (SIT) or high-intensity interval training (HIIT). SIT and HIIT methods involve repeated efforts between sets with recovery periods long enough to allow sprint capacity to recover almost completely. In contrast, recovery time between sprints in RST is minimal. Therefore, a decline in performance is inevitable in RST. A meta-analysis by Taylor et al. (1) reviewed controlled and uncontrolled trials examining the effects of RST on athletic performance variables, including the backward vertical jump, 10-, 20-, and 30-m sprints, repeated sprint capacity, and high-intensity intermittent running performance. The study ultimately concluded that RST effectively improves participants' strength, speed, repeated sprint capacity, and endurance.

Previous studies on RST have mostly focused on lower-body movement patterns. However, the upper limbs also play a crucial role in many sports. Due to having less muscle mass and a higher proportion of fast-twitch muscle fibres than the lower limbs, the physiological responses during upper-limb exercise may differ. For instance, peak oxygen consumption during upper-limb exercise is lower than during lower-limb exercise, and oxygen consumption kinetics are slower. This may be due to anatomical differences between the upper and lower limbs, such as the lower capillary density in the upper limbs, resulting in shorter blood transit times and reduced diffusion (2). Raphael et al. (3) found that VO_2_peak during seated upper-limb RST skiing accounted for only 65% of the athletes' running VO_2_max and that VO_2_max was not correlated with their RSA performance. Therefore, in this mode of exercise, peripheral rather than central cardiovascular factors may primarily be responsible for upper-limb recovery. Antunes et al. (4) investigated the correlation between upper-limb oxygen consumption, haemoglobin/myoglobin deoxygenation kinetics, and RSA in a group of judo athletes. The results showed that upper-limb oxygen consumption and [HHb] were not related to RSA. However, parameters such as maximum upper-limb aerobic power, peak VO_2_ during the upper-limb RSA test, and the first ventilatory threshold (VT_1_) were positively correlated with total work during RSA. This further supports the dominance of peripheral factors in upper-limb RSA. To date, no studies have examined the proportion of energy supply during upper-limb RSA, and further research is needed to elucidate the associated physiological responses.

In fact, RST-style training has a long history in boxing. Boxing coaches have widely adopted the RST model for their training programmes, based on the sport's characteristics and their own experience. However, research into the impact of RST interventions on boxing performance is limited. Kamandulis et al. (5) investigated the effects of a four-week punching bag RST programme on the punching power and upper-body aerobic capacity of well-trained boxers. The experimental group performed three sessions per week of sport-specific RST, and results demonstrated improvements in upper-body aerobic capacity and cumulative punching power during both maximal-effort and simulated sparring tests. Notably, lactate levels did not decrease following simulated matches despite improved aerobic capacity, raising the question of whether the observed benefits stemmed from enhanced anaerobic capacity or psychological factors. Given RST's physiological characteristics, it would be worthwhile to investigate whether sport-specific RST improves neuromuscular function in boxers.

Furthermore, boxing punching movements are whole-body movements. According to the “weakest link” theory, the magnitude of energy transfer is determined by the weakest link in the kinetic chain (6, 7). Therefore, it is anticipated that continuous punching movements may exert greater physiological stress on the upper limbs. Consequently, testing upper-limb anaerobic capacity and explosive power (upper-limb Wingate and punching capacity tests) will further our understanding of sport-specific RST in boxing. Simultaneously, assessing punching speed and performing the 30-s maximum punching test will help us to understand the impact of sport-specific RST on athletes' competitive performance.

Therefore, the purpose of this study was to investigate the effects of a 4-week boxing-specific repeated sprint training intervention on upper-body strength quality, anaerobic capacity, and punching ability in elite male boxers.

## 2 Methods

### 2.1 Participants

Sixteen male boxers from Wuhan Sports University voluntarily participated in this study. Their mean age was 20.63 ± 1.72 years, mean height was 175.00 ± 5.50 cm, and mean body mass was 72.25 ± 9.17 kg. All participants competed in national-level championships annually and were trained by the same coach. The participants were randomly assigned to either the experimental group (EG, *n* = 8) or the control group (CG, *n* = 8). To participate in the study, all boxers had to meet the following inclusion criteria: (1) at least 5 years of boxing training experience and a boxing rank of at least Class 1 athlete; (2) training at least three times per week; (3) free of injuries and neuromuscular issues for at least 10 weeks; and (4) not currently in a weight-cutting phase. All athletes and/or family members of athletes under 18 years of age were informed of the study objectives, associated benefits, experimental procedures, and potential risks prior to testing and intervention training and provided informed consent. This study was conducted in accordance with the Declaration of Helsinki.

Experimental procedure: Subject exclusion criteria: (1) subjects who missed three or more training sessions; (2) subjects who withdrew from the study due to injury or illness; and (3) subjects who were unable to complete the training intervention as required.

### 2.2 Study design

This study employed a randomized controlled design. Participants were randomly assigned using a computer-generated random number sequence. The allocation sequence was generated by an independent researcher not involved in participant recruitment or testing. Participants were stratified by baseline bench press MPP to ensure balanced groups. Sixteen participants were randomly assigned to either the EG or the CG for a 4-week training programme. The 4-week intervention duration was chosen based on previous sport-specific RST studies demonstrating that this timeframe is sufficient to induce meaningful neuromuscular and metabolic adaptations in combat athletes (8, 9). Training sessions were conducted three times per week during the intervention. These took place on three non-consecutive days (Monday, Wednesday, and Friday) from 10:30 a.m. to 12:00 p.m. Both groups were instructed to use the modified Borg CR-10 subjective fatigue scale (RPE 0–10) to control internal load during RST training. Prior to this, all coaches and athletes received training on the RPE 0–10 scale to familiarise themselves with its use. Each training session followed a standardised protocol (Table 1): it began with 20 min of dynamic stretching and a walking warm-up. Subsequently, all athletes performed 20 min of shadow boxing (RPE 5) and 25 min of technical sparring (RPE 5) under the supervision of experienced coaches, focusing on technical improvement. The EG group then performed three rounds of three sets of 14 repetitions (with a 10-s rest between sets, passive recovery during set rest, and a 1-min rest between rounds) of all-out, sport-specific (punching the heavy bag) interval training (RPE-10). This training volume (14 sets × 3 rounds) was selected based on previous work demonstrating that moderate-to-high volumes of repeated-sprint efforts are required to accumulate sufficient metabolic and neuromuscular stimulus for adaptation in trained athletes (5, 10). At the same time, the CG group performed three rounds of 3-min conventional punching bag technique training (RPE-5) with 1-min rest periods between rounds. Finally, all athletes performed 15 min of light punching and static stretching to cool down and conclude the training session. Pre- and post-tests were conducted for both groups on the third day before the intervention and on the third day after its completion.

**Table 1:** Standardised design of the intervention programme.

Training duration	Experimental group training content	Training intensity	Training duration	Control group training content	Training intensity
20 min	Dynamic stretching and warm-up while moving	—	20 min	Dynamic stretching and warm-up while moving	—
20 min	Shadowboxing	RPE-5	20 min	Shadowboxing	RPE-5
25 min	Technical training	RPE-5	25 min	Technical training	RPE-5
10 min	3 rounds of 3s × 14 sets (10s rest between sets, 1 min rest between rounds) All-out sport-specific (punching the heavy bag) interval training	RPE-10	10 min	3 rounds × 3 min (1 min rest between rounds) of standard heavy bag technique training	RPE-5
15 min	Relaxation shadowboxing and static stretching	—	15 min	Relaxation shadowboxing and static stretching	—

### 2.3 Experimental procedures

This study was conducted from 5 January to 5 February 2026. One week before testing began, the athletes completed a familiarisation session to minimise the learning effect. All assessments were scheduled between 10:00 and 12:00, with participants wearing the same attire each time, and were conducted by the same testers 48 h apart (on days three and five, before and after the intervention). The testers were unaware of the intervention details. All participants were instructed to: (1) ensure they got adequate sleep (more than eight hours) before each test; (2) maintain their usual dietary and hydration habits in the days leading up to the tests; (3) avoid consuming beverages containing stimulants, such as caffeine.

The testing procedure was completed within a single day, in the following order: bench press, punch speed, 30-s punch, and upper-body Wingate tests. Athletes were instructed to exert maximum effort during testing and to take adequate rest periods between each test. Prior to testing, a 15-min standardised warm-up consisting of low-intensity ergometer cycling, dynamic stretching, and low-intensity free bench presses was performed. For tests involving repeated measurements, the best single performance was selected for inclusion in the study data.

#### 2.3.1 Upper-body strength

The mean propulsive power (MPP) during the bench press (BP) is used to assess participants' upper-body strength levels. All tests were conducted on a Smith machine to eliminate the influence of horizontal displacement on the test results. Measurements were taken in manual mode using the My Jump 2 software (My Jump 2, version 2.0.3, developed by Carlos Balsalobre-Fernández), whose validity and reliability have been verified in numerous studies (11). For bench press assessment, the smartphone was fixed in a stationary position perpendicular to the barbell's path, and the application's manual frame-by-frame tracking mode was used to determine barbell velocity from which propulsive power was calculated. Although My Jump 2 was originally validated for vertical jump assessment, video-based motion analysis has been shown to provide valid measurements of barbell velocity during bench press exercise in prior research (12). This procedure ensured within-subject consistency for detecting changes over time. However, it is important to emphasize that the absolute MPP values reported herein have not been specifically validated against a gold-standard linear position transducer for bench press exercise; therefore, these values lack external validity and should not be directly compared with absolute power outputs reported in studies using force plates or linear position transducers. The data are interpretable only as relative indicators of pre-to-post change within this specific experimental setup. The My Jump 2 application was selected for this study because it provides a portable, cost-effective method for quantifying barbell velocity without the need for expensive laboratory equipment such as linear position transducers or force plates. Although this limits the external validity of absolute power values, the approach is consistent with the practical constraints of field-based research in applied sport settings, where access to gold-standard biomechanical instruments is often limited. Subjects were instructed to perform three repetitions at maximum speed at each load, starting with a load of 30% of body mass (BM) and increasing the load by 5% BM per set until a decline in MPP was observed (typically 2–5 sets), with a 1-min rest interval between sets, which is consistent with standard resistance training recommendations for power-oriented assessments, where rest intervals of 60–120 s are advised to allow sufficient ATP-PCr replenishment without full recovery, thereby maintaining neuromuscular readiness while minimizing fatigue accumulation (13). During the BP, the subject controlled the descent of the barbell until it lightly touched the chest, then moved the barbell as quickly as possible after the start command to achieve maximum average propulsion power. The start command was an auditory signal (“Go!”) delivered by the same tester for all participants and all trials. The My Jump 2 application's manual frame-by-frame tracking mode was synchronized with this auditory command, with velocity calculation initiated from the first video frame following the verbal cue. The maximum MPP achieved during the test was included in the experimental data.

#### 2.3.2 Upper-body anaerobic capacity

**Figure 1:**
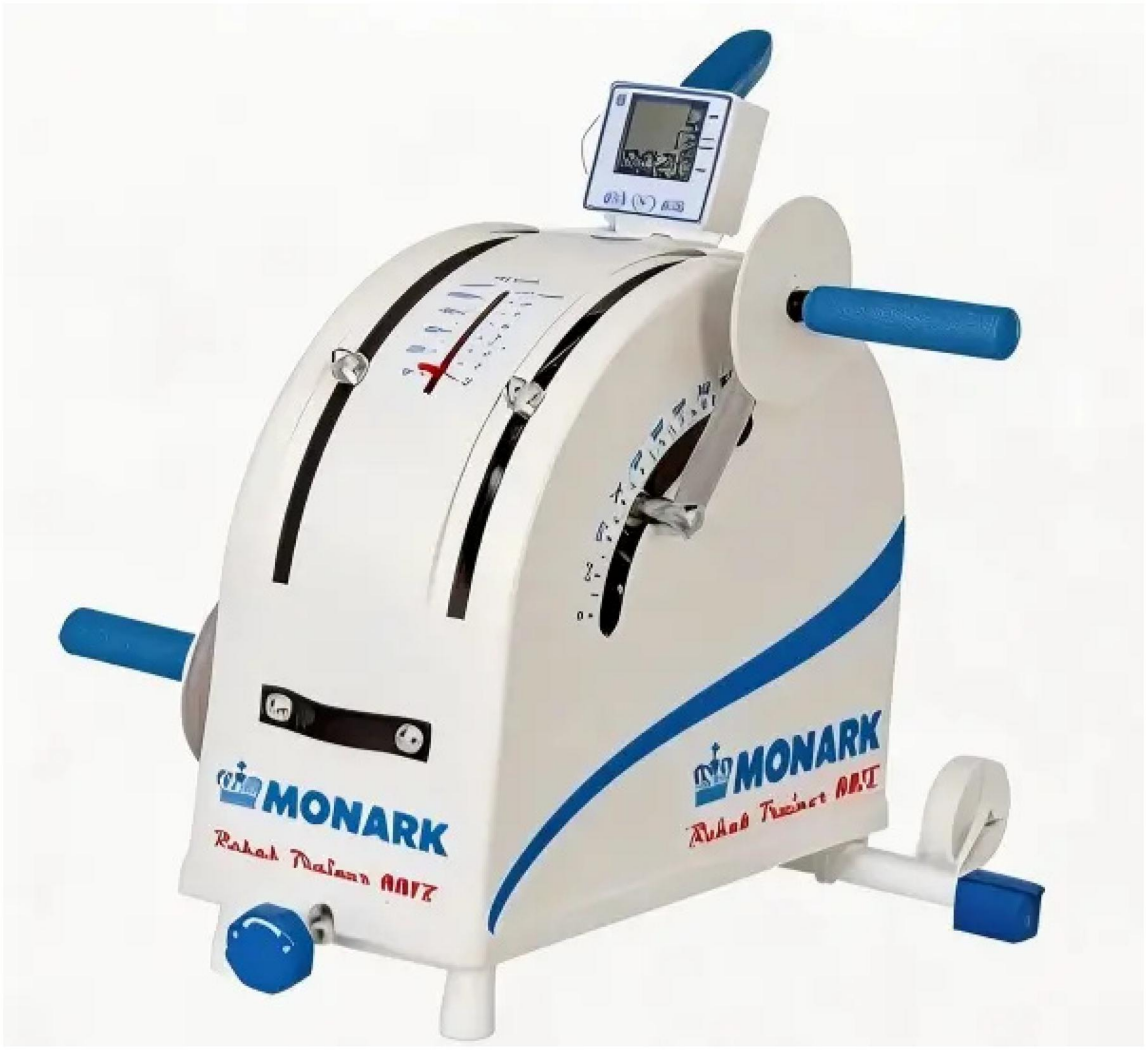
Monark894E upper-body wingate test cycle ergometer.

The Wingate test is a 30-s maximal-effort exercise performed at a constant load; it is the most commonly used test for assessing athletes' anaerobic performance on a cycle ergometer. The upper-body anaerobic capacity test was conducted using a Monark 894-E hand-cranked cycle ergometer (Figure 1), with the load set at 0.06 kg/kg (14). Participants were instructed to pedal the ergometer from a standstill at maximum effort for 30 s; the tester continuously encouraged participants throughout the test to ensure they exerted their utmost effort. Peak power (PP) and mean power (MP) over the 30 s were recorded and included in this study.

#### 2.3.3 Punching ability

**Figure 2:**
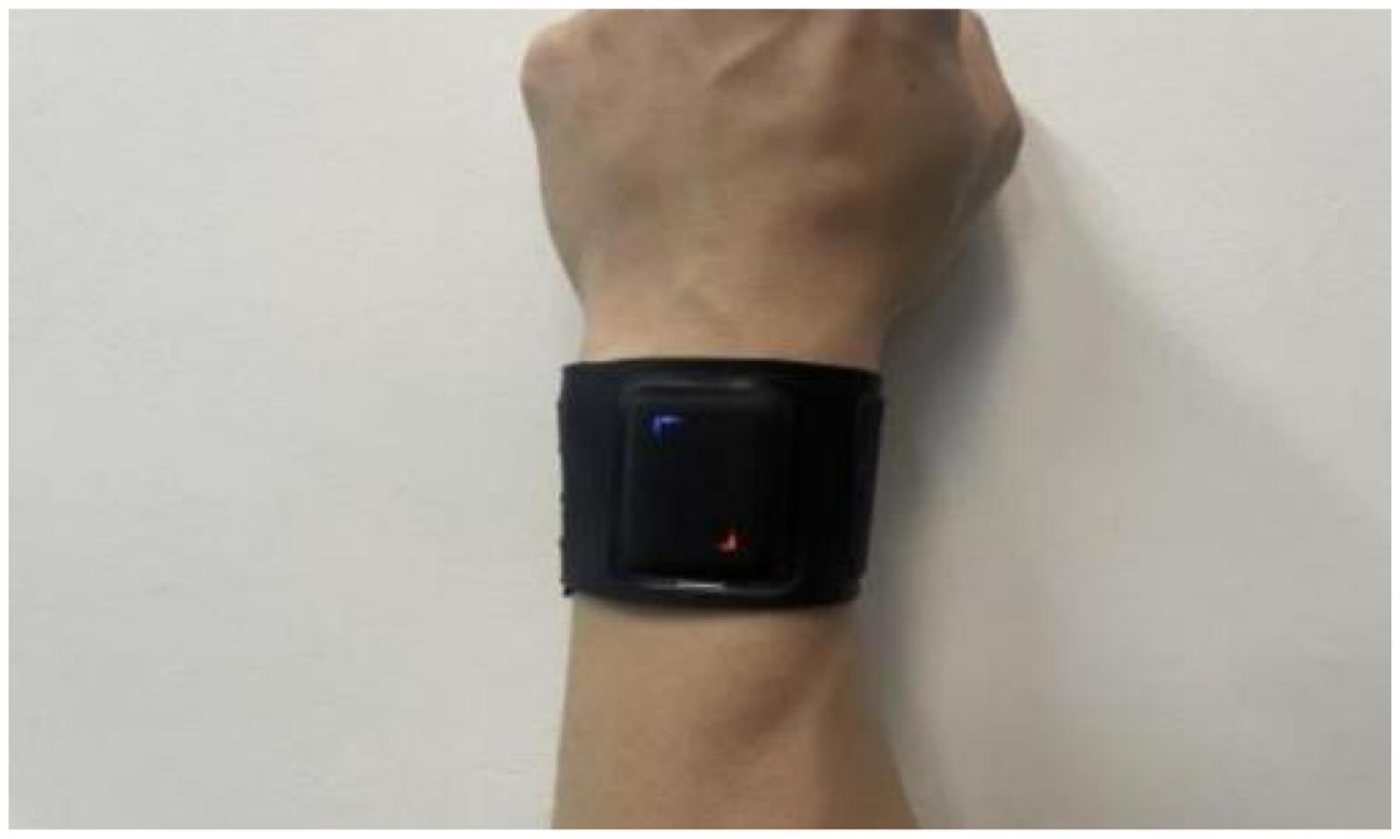
Corner boxing tracker.

The accurate and reliable assessment of kinematic data is crucial for monitoring training outcomes and sport-specific abilities. Quantifying punch kinematics is typically achieved using accelerometer-based inertial sensors and high-speed camera systems, which have been cross-validated. Moreover, with technological advancements, inertial sensors have been utilised to record various performance metrics related to boxing, such as punch velocity, mass, type, and frequency (15). Consequently, the present study employed the Corner Boxing Tracker (UK) (Figure 2) to quantify punch velocity and evaluate sport-specific performance, given its substantiated recognition accuracy (16). The inertial measurement unit (IMU) embedded in the Corner Boxing Tracker samples at a frequency of 200 Hz. Raw acceleration signals were filtered using a built-in low-pass Butterworth filter (cut-off frequency = 25 Hz) to attenuate high-frequency noise and motion artifacts, ensuring reliable punch velocity calculations. The striking target used in this study was a standardized boxing bag (Jiurishan).

Prior to testing, the sensor was secured to the subject's wrist extensor muscles using a specialised wrist strap. The subject then put on standard 3M boxing wrist wraps and AIBA-standard competition boxing gloves of the appropriate weight class.

First, a punch velocity test was conducted. Upon the tester's command, the subject struck the punching bag with a rear-hand straight punch at maximum speed. This test was repeated three times, and the fastest peak velocity (PV) from these trials was included in the study data. Subsequently, the subject struck the punching bag with maximum speed and effort for 30 s. To ensure experimental reliability, only straight punches were permitted. After exporting the data, the total number of punches (TP) and the average punch velocity (MV) were recorded as study data.

#### 2.3.4 Experimental control

1. Participants were instructed not to engage in any training other than the intervention training during the study period.
2. Participants were not to change their dietary or hydration habits during the intervention period.
3. Participants were to ensure adequate rest during the intervention period.
4. Throughout the intervention, experienced coaches provided motivation and supervision to the athletes to ensure that the prescribed training load was met.

### 2.4 Statistical analysis

Statistical analysis was performed using SPSS 27.0 (IBM Corp, Armonk, NY, USA), and data are presented as mean ± SD. The intra-class correlation coefficient (ICC) and coefficient of variation (CV) were used to assess the relative and absolute reliability of the MPP and punch speed tests; reliability was considered acceptable when ICC ≥ 0.8 and CV ≤ 10%.

Shapiro–Wilk tests were performed to verify data normality, and Levene's test was used to assess homogeneity of variances. An independent-samples *t*-test was conducted at baseline to verify successful randomization. A 2 × 2 repeated-measures ANOVA was then employed to examine the Time × Group interaction for each dependent variable. When a significant interaction was observed, simple effects tests were conducted to decompose the interaction.

Prior to the main hypothesis testing, the assumptions underlying the repeated-measures ANOVA were examined. The Shapiro–Wilk test confirmed that all dependent variables followed a normal distribution at both pre-test and post-test (all *p* > 0.05; Table 2). Independent samples *t*-tests verified successful randomization, with no significant baseline differences between the experimental and control groups on any outcome measure (all *p* > 0.05; Table 3). Levene's test indicated homogeneity of variances across groups for all variables (all *p* > 0.05; Table 4). As the within-subject factor (time) comprised only two levels (pre- and post-intervention), the assumption of sphericity was not applicable.

**Table 2:** Results of the normality test.

Category	Time	Group	Sample size	Mean	SD	Skewness	Kurtosis	Kolmogorov–Smirnov test		Shapiro–Wilk test	
								Statistc *D*	*p* value	Statistc *W*	*p* value
MPP (W)	Pre-test	CG	8	346.82	64.231	1.664	3.496	0.261	0.116	0.853	0.102
		EG	8	340.546	62.113	0.024	−2.101	0.227	0.269	0.874	0.166
	Post-test	CG	8	347.695	62.992	1.783	3.805	0.267	0.095	0.83	0.059
		EG	8	409.921	58.156	−0.042	−1.984	0.244	0.19	0.887	0.218
PP (W)	Pre-test	CG	8	442.125	38.621	0.993	1.447	0.235	0.228	0.888	0.224
		EG	8	454.125	69.608	−0.375	−1.07	0.172	0.692	0.945	0.657
	Post-test	CG	8	441.5	37.959	0.965	1.164	0.172	0.696	0.915	0.39
		EG	8	530.125	68.691	−0.414	−1.337	0.21	0.391	0.893	0.25
MP (W)	Pre-test	CG	8	344.809	37.436	−0.589	0.937	0.155	0.821	0.967	0.873
		EG	8	358.064	65.039	0.664	−0.593	0.233	0.234	0.927	0.485
	Post-test	CG	8	344.875	39.718	−0.566	0.165	0.132	0.951	0.979	0.96
		EG	8	431.939	64.414	0.812	−0.801	0.297	0.039	0.878	0.179
PV (m/s)	Pre-test	CG	8	10.901	0.985	−0.478	−1.32	0.217	0.341	0.924	0.46
		EG	8	10.444	0.804	−0.176	−0.586	0.137	0.932	0.967	0.873
	Post-test	CG	8	10.901	0.896	−0.369	−1.458	0.226	0.276	0.917	0.407
		EG	8	11.204	0.512	0.317	−1.351	0.171	0.703	0.944	0.653
TP (times)	Pre-test	CG	8	169.875	26.335	0.499	1.869	0.225	0.279	0.944	0.649
		EG	8	172	24.617	−0.584	−0.772	0.19	0.545	0.913	0.375
	Post-test	CG	8	177	23.755	0.234	1.384	0.227	0.264	0.956	0.767
		EG	8	200.875	25.609	−0.301	−0.837	0.159	0.794	0.951	0.721
MV (m/s)	Pre-test	CG	8	8.259	1.328	0.935	2.013	0.193	0.517	0.942	0.631
		EG	8	7.976	0.919	−0.683	−0.786	0.197	0.484	0.915	0.388
	Post-test	CG	8	8.277	1.21	0.638	1.941	0.193	0.512	0.946	0.67
		EG	8	9.164	0.838	−0.653	−1.052	0.273	0.081	0.861	0.123

**Table 3:** Results of the independent samples t-test.

Category	Group (mean ± SD)		*t*	*p*
	CG (*n* = 8)	EG (*n* = 8)		
MPP (W)	346.820 ± 64.23	340.55 ± 62.11	0.199	0.845
PP (W)	442.13 ± 38.62	454.13 ± 69.61	−0.426	0.676
MP (W)	344.81 ± 37.44	358.06 ± 65.04	−0.500	0.625
PV (m/s)	10.90 ± 0.99	10.44 ± 0.80	1.017	0.326
TP (times)	169.88 ± 26.34	172.00 ± 24.62	−0.167	0.870
MV (m/s)	8.26 ± 1.33	7.98 ± 0.92	0.495	0.628

**Table 4:** Results of the ANOVA.

Category	Variance by group (SD)				*F*	*p*
	EG pre-test (*n* = 8)	CG pre-test (*n* = 8)	EG pro-test (*n* = 8)	CG pro-test (*n* = 8)		
MPP (W)	62.11	64.23	58.16	62.99	0.288	0.834
PP (W)	69.61	38.62	68.69	37.96	1.967	0.142
MP (W)	65.04	37.44	64.41	39.72	2.560	0.075
PV (m/s)	0.80	0.99	0.51	0.90	1.550	0.224
TP (times)	24.62	26.34	25.61	23.75	0.074	0.973
MV (m/s)	0.92	1.33	0.84	1.21	0.131	0.941

#### 2.4.1 Control for multiple comparisons

To control for the inflation of Type I error arising from multiple *post-hoc* comparisons, a Bonferroni correction was applied to the significance level of the simple effects tests. Specifically, for each significant Time × Group interaction, the alpha level for the subsequent four simple effect comparisons (between-group at pre-test, between-group at post-test, within-group for CG, within-group for EG) was adjusted to *α*_adjusted = 0.05/4 = 0.0125. Baseline between-group differences were assessed using independent *t*-tests with an uncorrected *α* = 0.05, as these were planned orthogonal comparisons conducted solely for verification of randomization and did not constitute part of the primary hypothesis testing.

#### 2.4.2 Effect size reporting

Given the relatively small sample size (*n* = 16), effect sizes were computed and reported alongside *p*-values to facilitate interpretation of practical significance. For repeated-measures ANOVA, partial eta-squared (*η_p_*^2^) is reported as a measure of variance explained, with thresholds of 0.01 (small), 0.06 (medium), and 0.14 (large). For within-group and between-group comparisons, Cohen's *d* was calculated using the pooled standard deviation and interpreted according to the following benchmarks proposed by Cohen and further refined by Hopkins et al. for sports science research: trivial (<0.2), small (0.2–0.6), moderate (0.6–1.2), large (1.2–2.0), and very large (>2.0) (17). Cohen's *d* reflects the standardized magnitude of the difference between two means, independent of sample size, thereby providing an estimate of the practical relevance of the observed effect. The 95% confidence intervals for Cohen's *d* were computed using the non-central t-distribution method to provide an estimate of precision.

#### 2.4.3 *Post-hoc* power analysis

Given the relatively small sample size dictated by the availability of elite-level boxers, a *post-hoc* power analysis was conducted to evaluate the sensitivity of the observed interaction effects. Using G*Power software (Version 3.1), we computed the achieved power for the Time × Group interaction in the 2 × 2 repeated-measures ANOVA. Based on the large effect sizes observed for MPP (*η_p_*^2^ = 0.985) and Wingate Mean Power (*η_p_*^2^ = 0.973), the achieved statistical power exceeded 0.99. However, for outcomes with moderate effects (e.g., Peak Punch Velocity, *η_p_*^2^ = 0.457), the observed power was 0.68. Consequently, while the detection of large interaction effects was robust, the study may be underpowered to detect smaller, potentially meaningful changes. The absence of an *a priori* power calculation is acknowledged as a limitation that increases the risk of Type II error for secondary outcomes.

The statistical significance level was set at *p* < 0.05 (or adjusted *p* < 0.0125 for simple effects following significant interactions).

## 3 Results

### 3.1 Upper-body strength

For the MPP of upper limb BP, the results of the repeated measures ANOVA showed that the main effect of group was not significant (*F* = 0.818, *p* = 0.381, partial *η*^2^ = 0.055); the main effect of time was significant (*F* = 984.376, *p* < 0.001, partial *η*^2^ = 0.986); and the interaction effect between measurement time and group was significant (*F* = 935.943, *p* < 0.001, partial *η*^2^ = 0.985).

The results of simple effects tests showed that: in the pre-intervention assessment, the main effect of group was not significant (*t* = −0.199, *p* = 0.845); in the post-intervention assessment, the between-group difference did not reach the conventional threshold of statistical significance (*t* = −2.053, *p* = 0.059). However, the magnitude of the difference corresponded to a large effect size [Cohen's *d* = 1.03, 95% CI (−0.04, 2.07)], indicating a potentially meaningful improvement that warrants further investigation in a larger sample. In the control group, the main effect of pre- and post-tests was not significant [*t* = −0.553, *p* = 0.589, Cohen's *d* = 0.01, 95% CI (−0.97, 0.99)]; in the experimental group, the main effect of pre- and post-tests was significant [*t* = −43.818, *p* < 0.001, Cohen's *d* = 1.15, 95% CI (0.07, 2.20)], indicating a significant increase in MPP following the intervention. Specific details are shown in Table 5.

**Table 5:** Data table for repeated-measures ANOVA of MPP with effect sizes.

MPP (W)	Pre-test (M ± SD)	Post-test (M ± SD)	ANOVA effects	*F*	*p*	Partial *η*^2^	Cohen's *d* within [95% CI]	Cohen's *d* between [95% CI]
CG (*n* = 8)	346.820 ± 64.231	347.695 ± 62.992	—	—	—	—	0.01 [−0.97, 0.99]	—
EG (*n* = 8)	340.546 ± 62.113	409.921 ± 58.156	—	—	—	—	1.15 [0.07, 2.20]	—
Group	—	—	Group	0.818	0.381	0.055	—	—
Time	—	—	Time	984.376	0.000	0.986	—	—
Group × Time	—	—	Group × Time	935.943	0.000	0.985	—	1.03 [−0.04, 2.07] (large)

### 3.2 Upper-body anaerobic capacity

For peak power (PP) in the upper-limb Wingate test, the results of the repeated-measures analysis of variance showed that the main effect of group was not significant (*F* = 3.254, *p* = 0.093, partial *η*^2^ = 0.189); the main effect of time was significant (*F* = 461.182, *p* < 0.001, partial *η*^2^ = 0.971); and the interaction effect between measurement time and group was significant (*F* = 476.605, *p* < 0.001, partial *η*^2^ = 0.971).

The results of the simple effects test showed that, at the pre-intervention assessment, the main effect of group was not significant (*t* = −0.426, *p* = 0.676). In the post-intervention assessment, the main effect of group was significant [*t* = −3.194, *p* = 0.006, Cohen's *d* = 1.60, 95% CI (0.41, 2.74)], with the EG's PP significantly higher than that of the CG. In the control group, the main effect of time was not significant [*t* = 0.252, *p* = 0.805, Cohen's *d* = −0.02, 95% CI (−1.00, 0.97)]; in the experimental group, the main effect of time was significant [*t* = −30.622, *p* < 0.001, Cohen's *d* = 1.10, 95% CI (0.03, 2.14)], and PP significantly increased after the intervention. Specific details are shown in Table 6.

**Table 6:** Data table for repeated-measures ANOVA of PP with effect sizes.

PP (W)	Pre-test (M ± SD)	Post-test (M ± SD)	ANOVA effects	*F*	*p*	Partial *η*^2^	Cohen's *d* within [95% CI]	Cohen's *d* between [95% CI]
CG (*n* = 8)	442.125 ± 38.621	441.500 ± 37.959	—	—	—	—	−0.02 [−1.00, 0.97]	—
EG (*n* = 8)	454.125 ± 69.608	530.125 ± 68.691	—	—	—	—	1.10 [0.03, 2.14]	—
Group	—	—	Group	3.254	0.093	0.189	—	—
Time	—	—	Time	461.182	0.000	0.971	—	—
Group × Time	—	—	Group × Time	476.605	0.000	0.971	—	1.60 [0.41, 2.74] (large)

For mean power (MP) in the upper-limb Wingate test, the results of the repeated-measures analysis of variance showed that the main effect of group was not significant (*F* = 3.558, *p* = 0.080, partial *η*^2^ = 0.203); the main effect of time was significant (*F* = 505.984, *p* < 0.001, partial *η*^2^ = 0.973); and the interaction effect between measurement time and group was significant (*F* = 504.172, *p* < 0.001, partial *η*^2^ = 0.973). Results from simple effects tests showed that at the pre-intervention measurement, the main effect of group was not significant (*t* = −0.500, *p* = 0.625). In the post-intervention test, the main effect of group was significant [*t* = −3.254, *p* = 0.006, Cohen's *d* = 1.61, 95% CI (0.42, 2.75)], with the MP in the EG significantly higher than that in the CG. In the control group, the main effect of time was not significant [*t* = −0.029, *p* = 0.978, Cohen's *d* = 0.00, 95% CI (−0.98, 0.98)]; in the experimental group, the main effect of time was significant [*t* = −31.783, *p* < 0.001, Cohen's *d* = 1.14, 95% CI (0.06, 2.19)], and MP significantly increased after the intervention. Specific details are shown in Table 7.

**Table 7:** Data table for repeated-measures ANOVA of MP with effect sizes.

MP (W)	Pre-test (M ± SD)	Post-test (M ± SD)	ANOVA effects	*F*	*p*	Partial *η*^2^	Cohen's *d* within [95% CI]	Cohen's *d* between [95% CI]
CG (*n* = 8)	344.809 ± 37.436	344.875 ± 39.718	—	—	—	—	0.00 [−0.98, 0.98]	—
EG (*n* = 8)	358.064 ± 65.039	431.939 ± 64.414	—	—	—	—	1.14 [0.06, 2.19]	—
Group	—	—	Group	3.558	0.080	0.203	—	—
Time	—	—	Time	505.984	0.000	0.973	—	—
Group × Time	—	—	Group × Time	504.172	0.000	0.973	—	1.61 [0.42, 2.75] (large)

### 3.3 Punching ability

For peak velocity (PV) in the punch speed test, the results of the repeated measures analysis of variance (ANOVA) showed that the main effect of group was not significant (*F* = 0.039, *p* = 0.847, partial *η*^2^ = 0.003); the main effect of time was significant (*F* = 11.781, *p* = 0.004, partial *η*^2^ = 0.457); and the interaction effect between measurement time and group was significant (*F* = 11.781, *p* = 0.004, partial *η*^2^ = 0.457). Results from simple effects tests showed that in the pre-intervention test, the main effect of group was not significant (*t* = 1.017, *p* = 0.326); in the post-intervention test, the main effect of group was not significant [*t* = −0.829, *p* = 0.421, Cohen's *d* = 0.41, 95% CI (−0.58, 1.39)]. In the control group, the main effect of time was not significant [*t* = 0.000, *p* = 1.000, Cohen's *d* = 0.00, 95% CI (−0.98, 0.98)]; in the experimental group, the main effect of time was significant [*t* = −4.854, *p* < 0.001, Cohen's *d* = 1.13, 95% CI (0.05, 2.18)], and PV significantly increased after the intervention, though the between-group difference was not significant (*p* = 0.421) and should be interpreted cautiously. Specific details are shown in Table 8.

**Table 8:** Data table for repeated-measures ANOVA of PV with effect sizes.

PV (m/s)	Pre-test (M ± SD)	Post-test (M ± SD)	ANOVA effects	*F*	*p*	Partial *η*^2^	Cohen's *d* within [95% CI]	Cohen's *d* between [95% CI]
CG (*n* = 8)	10.901 ± 0.985	10.901 ± 0.896	—	—	—	—	0.00 [−0.98, 0.98]	—
EG (*n* = 8)	10.444 ± 0.804	11.204 ± 0.512	—	—	—	—	1.13 [0.05, 2.18]	—
Group	—	—	Group	0.039	0.847	0.003	—	—
Time	—	—	Time	11.781	0.004	0.457	—	—
Group × Time	—	—	Group × Time	11.781	0.004	0.457	—	0.41 [−0.58, 1.39] (small)

For the total number of punches (TP) in the 30-s punching test, the results of the repeated measures ANOVA showed that the main effect of group was not significant (*F* = 1.080, *p* = 0.316, partial *η*^2^ = 0.072); the main effect of time was significant (*F* = 310.486, *p* < 0.001, partial *η*^2^ = 0.957); and the interaction effect between measurement time and group was significant (*F* = 113.333, *p* < 0.001, partial *η*^2^ = 0.890). Results of simple effects tests showed: In the pre-intervention assessment, the main effect of group was not significant (*t* = −0.167, *p* = 0.870); in the post-intervention assessment, the between-group comparison was not statistically significant (*t* = −1.933, *p* = 0.074). Nevertheless, the observed effect was large [Cohen's *d* = 0.97, 95% CI (−0.08, 2.00)], suggesting that the lack of significance may reflect insufficient statistical power rather than the absence of a training effect. In the control group, the main effect of time was significant [*t* = −4.932, *p* < 0.001, Cohen's *d* = 0.28, 95% CI (−0.71, 1.27)], and TP significantly improved after the intervention; in the experimental group, the main effect of time was significant [*t* = −19.987, *p* < 0.001, Cohen's *d* = 1.15, 95% CI (0.07, 2.20)], and TP significantly improved after the intervention. Specific details are shown in Table 9.

**Table 9:** Data table for repeated-measures ANOVA of TP with effect sizes.

TP (times)	Pre-test (M ± SD)	Post-test (M ± SD)	ANOVA effects	*F*	*p*	Partial *η*^2^	Cohen's *d* within [95% CI]	Cohen's *d* between [95% CI]
CG (*n* = 8)	169.875 ± 26.335	177.000 ± 23.755	—	—	—	—	0.28 [−0.71, 1.27]	—
EG (*n* = 8)	172.000 ± 24.617	200.875 ± 25.609	—	—	—	—	1.15 [0.07, 2.20]	—
Group	—	—	Group	1.080	0.316	0.072	—	—
Time	—	—	Time	310.486	0.000	0.957	—	—
Group × Time	—	—	Group × Time	113.333	0.000	0.890	—	0.97 [−0.08, 2.00] (large)

For the mean punch velocity (MV) in the 30 s punch test, the results of the repeated measures ANOVA showed that the main effect of group was not significant (*F* = 0.309, *p* = 0.587, partial *η*^2^ = 0.022); the main effect of time was significant (*F* = 92.758, *p* < 0.001, partial *η*^2^ = 0.869); and the interaction effect between measurement time and group was significant (*F* = 87.080, *p* < 0.001, partial *η*^2^ = 0.861). Results of simple effects tests showed: In the pre-intervention assessment, the main effect of group was not significant (*t* = −0.495, *p* = 0.628); in the post-intervention assessment, the main effect of group was not significant [*t* = −1.703, *p* = 0.111, Cohen's *d* = 0.85, 95% CI (−0.18, 1.87)]. In the control group, the main effect of time was not significant [*t* = −0.212, *p* = 0.835, Cohen's *d* = 0.01, 95% CI (−0.97, 0.99)]; in the experimental group, the main effect of time was significant [t = −13.409, *p* < 0.001, Cohen's *d* = 1.35, 95% CI (0.24, 2.43)], and MV significantly increased after the intervention, the between-group difference was not significant (*p* = 0.111) and should be interpreted cautiously. Specific details are shown in Table 10.

**Table 10:** Data table for repeated-measures ANOVA of MV with effect sizes.

MV (m/s)	Pre-test (M ± SD)	Post-test (M ± SD)	ANOVA effects	*F*	*p*	Partial *η*^2^	Cohen's *d* within [95% CI]	Cohen's *d* between [95% CI]
CG (*n* = 8)	8.259 ± 1.328	8.277 ± 1.210	—	—	—	—	0.01 [−0.97, 0.99]	—
EG (*n* = 8)	7.976 ± 0.919	9.164 ± 0.838	—	—	—	—	1.35 [0.24, 2.43]	—
Group	—	—	Group	0.309	0.587	0.022	—	—
Time	—	—	Time	92.758	0.000	0.869	—	—
Group × Time	—	—	Group × Time	87.080	0.000	0.861	—	0.85 [−0.18, 1.87] (large)

## 4 Discussion and analysis

### 4.1 Upper-body strength

Following the intervention, the experimental group demonstrated a meaningful improvement in bench press power output, with large effect sizes observed both within and between groups, despite the between-group difference not reaching statistical significance. This is the first study to investigate the effects of a boxing-specific RST programme on upper-body explosive power. Prior to this study, no research had examined the impact of RST on upper-body strength, whereas studies on lower-body RST had demonstrated improvements in muscle strength.

This study provides the first evidence of the positive effects of upper-body RST on muscle strength, suggesting that upper-body repeated sprints have a similar adaptive potential to lower-body exercises. Regarding studies on sport-specific RST for combat athletes, these findings are consistent with previous research demonstrating that short-term RST, involving specialised techniques, can significantly enhance lower-body concentric explosive strength (SJ) in taekwondo and karate athletes. This suggests that sport-specific RST has promising applications.

While the precise physiological mechanisms by which RST enhances neuromuscular function remain to be fully elucidated, insights may be gained from research on HIIT, which follows similar training principles. Human muscle strength is primarily influenced by “myogenic” and “neurogenic” factors. Studies using high-density electromyography (EMG) have found that high-intensity interval running increases muscle fibre conduction velocity, maximum knee extension torque, and the firing rate of high-threshold motor units, all of which are closely related to the generation of maximum force (18). In contrast, only minor changes in these functional indicators were observed following moderate-duration exercise interventions. Recent studies indicate that HIIT, whether performed alone or in combination with increased protein utilisation through higher protein intake, promotes skeletal muscle anabolism (19). Short-term high-intensity interval training increases muscle protein synthesis (MPS) rates, and new evidence from omics-based approaches suggests that high-intensity interval exercise upregulates unique gene signatures and proteins associated with anabolic signalling. While high-intensity interval training does not increase muscle fibre cross-sectional area (CSA) or the number of satellite cells, it is associated with increases in total muscle (i.e., muscle CSA/volume as measured by MRI) and whole-body (i.e., lean body mass as measured by dual-energy x-ray absorptiometry) across different age groups (20). Both of these are markers of muscle hypertrophy. Changes in neuromuscular factors and muscle fibre CSA are directly related to increases in muscle strength. Furthermore, Halperin et al. (21) found that repeated sprint exercise elicits similar physiological and perceptual responses in the upper and lower limbs. This suggests that upper-limb repeated sprint training (RST) may produce adaptations similar to those in the lower limbs. Taken together, this evidence suggests that the potential adaptive mechanisms—while necessarily speculative in the absence of direct physiological measures—may include increased muscle fibre conduction velocity, discharge rate of high-threshold motor units, and muscle fibre CSA. However, we acknowledge that direct measures (e.g., EMG, muscle biopsies) were not collected in the present study; therefore, these mechanistic interpretations remain inferential and require direct verification in future research.

For boxers, strength qualities are highly correlated with athletic performance. Studies have shown a strong correlation between upper- and lower-limb strength and punch speed and power in boxers (22). The correlation coefficient between the MPP used in this study and all punching techniques ranged from 0.7 to 0.79, and athletes with higher strength levels threw more punches during competitions. Therefore, sport-specific RST can be an effective training method for boxing.

### 4.2 Upper-body anaerobic capacity

The 4-week boxing-specific RST programme produced substantial improvements in upper-limb Wingate peak and mean power, with large effect sizes observed both within and between groups. This indicates that this type of training positively affects boxing athletes' maximum power output and anaerobic endurance. As early as 1998, MacDougall et al. (23) found that relatively brief but intense sprint training could increase glycolytic enzyme activity and maximum short-term power output. In the same year, Dawson et al. (24) investigated the effects of short-distance sprints on athletic performance, muscle metabolism, and fibre type, finding that six weeks of short-distance sprinting improved trainees' anaerobic endurance, increased phosphokinase activity, and raised the proportion of type II muscle fibres. Subsequently, Ørtenblad et al. (25) found that short-distance sprint training improved sarcoplasmic reticulum function, thereby enhancing calcium ion release. Consequently, the implementation of repeated sprint training employing short-distance sprints may result in analogous training adaptations. This assertion is corroborated by the findings of Zinner et al. (2), who observed that six sessions of sprint interval training over a period of 11 days resulted in an augmentation of both peak and average power output in the upper and lower limbs during the Wingate test. Moreover, several studies on repeated lower-limb sprint training have reported similar results (26). This finding indicates that repeated sprint training can enhance anaerobic capacity, potentially through improvements in neuromuscular function and increased activity of phosphorylase and glycolytic enzymes. Although direct enzymatic or metabolic measurements were beyond the scope of this investigation, the substantial improvements in Wingate power observed herein are consistent with these previously documented adaptations. The findings of this study demonstrate that boxing-specific RST is adequate to generate stimuli analogous to conventional RST, thereby achieving the objective of enhancing anaerobic capacity. Khanna et al. (27) conducted a study of the physiological and biochemical characteristics of Indian national boxing athletes. Their findings indicated that peak blood lactate concentrations in advanced boxers were significantly higher than those in novice boxers. This suggests that anaerobic endurance has a substantial impact on the level of boxing training. In summary, anaerobic capacity is a crucial component of a boxer's competitive ability, and the training effects of sport-specific RST will improve a boxer's competitive performance.

### 4.3 Punching ability

The experimental group exhibited meaningful within-group improvements across all punching performance variables, with large effect sizes observed for peak punch velocity, total punches, and mean punch velocity. Although between-group differences for total punch count and punch velocities did not reach statistical significance, the associated effect sizes suggested practically meaningful enhancements. Numerous studies have demonstrated the positive effects of RST on sport-specific performance. In the field of combat sports, Ouergui et al. (28) examined the effect of sport-specific RST on the performance of taekwondo athletes. The findings demonstrated a substantial enhancement in the taekwondo-specific Frequency-Speed Kick Test (FSKT) following a 4-week intervention period. Ouergui et al. (9) conducted a 4-week sport-specific RST intervention on 36 adolescent taekwondo athletes. Compared with the control group, the experimental group performed more kicks in the 10 s sport-specific test and achieved higher scores on the Taekwondo Specific Agility Test (TSAT).

In summary, given the potential adaptations of repeated sprint training to aerobic, anaerobic, and neuromuscular performance, this study examined whether short-term boxing-specific RST can improve punch velocity, total punches, and average punch velocity during a 30-s punch test in boxing athletes. The findings showed that following the intervention, the experimental group showed significant improvements in peak punching speed (*d* = 1.13), total number of punches (*d* = 1.15), and average punching speed (*d* = 1.35). In contrast, the control group exhibited a small increase in total punches (*d* = 0.28). The between-group difference in TP post-intervention did not reach statistical significance (*p* = 0.074) but corresponded to a large effect size (*d* = 0.97), suggesting that the training effect was meaningful despite limited statistical power.

Regarding the variables of peak punch speed and average punch speed, the between-group differences at post-test did not reach statistical significance (PV: *p* = 0.421; MV: *p* = 0.111). However, the within-group improvements in the experimental group were associated with large effect sizes for both PV (*d* = 1.13) and MV (*d* = 1.35), whereas the control group showed negligible changes (PV: *d* = 0.00; MV: *d* = 0.01). These data suggest that sport-specific RST may positively influence punch velocity, although larger samples are needed to detect between-group differences with adequate statistical power.

Despite the widely held view that speed is a genetically determined attribute with limited capacity for enhancement through training (29), the findings of this study (a 7.3% increase in maximum punch speed in the experimental group, *d* = 1.13) suggest that sport-specific repeated sprint training may have a positive effect on punch speed in boxers. As indicated by previous literature, the implementation of a repeated sprint training (RST) programme has been demonstrated to enhance acceleration, as evidenced by studies reporting increases in 10-m sprint times and 20-m sprint speeds (30). It has been reported that RST programs have a positive and significant effect on SJ and CMJ performance in the training group. This may indicate improvements in the force-velocity curve, force-time characteristics, or SSC contraction capacity of the leg extensors. Improvements in the CMJ also reflect an enhanced ability to utilise stored elastic energy (similar to the RSA) and indirectly support the first phase of the force-time curve in the training group. This is driven by the rate of force development (RFD) of the leg extensors during the initial 180–250 ms. Research indicates that improvements in any one of speed, jumping power, or strength influence one another (31), which may further explain the CMJ improvements observed in previous studies. Indeed, the temporal demands of punching movements are lower than those of lower-body jumping movements, yet they require greater force over a shorter timeframe. Consequently, RST may have enhanced the force-velocity curves and force-time characteristics of upper-body muscles, thereby facilitating increased punching speed.

Regarding the outcomes of the 30-s punching test, the experimental group showed a large within-group increase in total punches (*d* = 1.15) and mean punch velocity (*d* = 1.35). A series of experiments utilising diverse dynamometers has demonstrated that the assessment of anaerobic capacity, founded upon total power output during the initial 30 s of maximal exercise, exhibits reliability. Although a formal correlation analysis was not conducted, the observed increase in total punches over 30 s appears to reflect, at least in part, an enhancement in anaerobic endurance. The potential adaptive mechanisms may include elevated lactate tolerance, augmented glycolytic enzyme activity, and increased energy substrate reserves. Conversely, the augmented total number of punches thrown may be ascribed to enhanced biomechanical efficiency, which is concomitant with increased muscle explosiveness, improved motor unit synchronization, and augmented stretch-shortening cycle efficiency.

Furthermore, the possibility that the observed Wingate power improvements simply reflect motor learning or familiarization with the test procedure warrants consideration. Several aspects of the experimental design argue against this interpretation. First, all participants completed a dedicated familiarization session one week prior to baseline testing, during which they practiced the Wingate test and punching assessments under maximal effort conditions. Second, the control group underwent identical pre- and post-testing procedures but showed negligible changes in Wingate peak power (*d* = −0.02) and mean power (*d* = 0.00) over the 4-week period. If systematic learning effects were operative, similar improvements would be expected in both groups. Third, the Wingate test involves a simple, repetitive cranking motion with minimal coordinative complexity; learning effects, if present, typically plateau after a single exposure. Therefore, the large and selective improvements observed exclusively in the experimental group are unlikely to be attributable to motor learning and instead reflect genuine physiological adaptation to the RST stimulus.

Finally, although no comparative analysis was conducted, it is hypothesised that training methods with similar movement patterns and energy metabolism characteristics are more beneficial for sport-specific performance. Therefore, it is recommended that sport-specific RST be implemented to enhance boxers' sport-specific abilities.

### 4.4 Interpretation of physiological mechanisms in the absence of direct measurements

A legitimate question arising from the present findings is whether the observed improvements can be confidently attributed to neuromuscular and metabolic adaptations, given that no direct physiological measurements (e.g., EMG, muscle biopsy, enzyme assays) were collected. While we acknowledge this limitation, several lines of indirect evidence support this interpretation. First, the 4-week intervention duration is too brief to induce detectable muscle hypertrophy; therefore, the substantial increases in upper-body strength and power—reflected by large within-group effect sizes for MPP (*d* = 1.15) and Wingate peak power (*d* = 1.10)—are almost certainly neural in origin. Early strength gains are consistently attributed to enhanced motor unit recruitment, increased firing frequency, and reduced antagonist co-activation. Second, previous studies employing similar repeated-sprint protocols with direct muscle sampling have documented increased activity of glycolytic enzymes (e.g., phosphofructokinase) and enhanced sarcoplasmic reticulum Ca^2^⁺ release. The magnitude and direction of the Wingate power improvements observed herein are fully consistent with these documented adaptations. Third, the specificity of the improvements—i.e., gains were observed in all-out anaerobic tasks (Wingate, 30 s punching) but were less pronounced in single-effort peak velocity—aligns with the expected profile of metabolic rather than purely coordinative adaptation. Collectively, while direct physiological confirmation remains a goal for future research, the pattern, time course, and magnitude of the observed changes provide indirect but converging evidence consistent with the hypothesis that neural and glycolytic adaptations underpin the performance enhancements reported here. However, without direct measures, these mechanistic interpretations remain provisional.

## 5 Conclusions

This study demonstrated that a 4-week boxing-specific repeated sprint training programme, incorporated into the conventional training of elite male boxers, substantially enhanced upper-body anaerobic capacity and produced preliminary evidence of improvements in explosive strength and punching endurance, although between-group differences in punching outcomes did not reach statistical significance and require confirmation in larger samples. Given the brief intervention duration, the observed adaptations are primarily attributable to neural and metabolic mechanisms rather than structural hypertrophy. These findings support the integration of sport-specific RST into the pre-competition training phase as a time-efficient strategy to augment upper-body performance. However, given the small sample size and the acknowledged measurement limitations, these findings should be considered preliminary and interpreted cautiously. Future research with larger samples, direct physiological measurements, and longer intervention periods is warranted to confirm these preliminary observations and to explore the underlying mechanisms more definitively.

## 6 Limitations

Several methodological considerations should be noted when interpreting these findings.

(1) The sample was limited to 16 elite male boxers due to the restricted availability of national-level athletes. The absence of an *a priori* power calculation and the consequent risk of Type II error should be considered when evaluating non-significant between-group comparisons (e.g., MPP, *p* = 0.059; TP, *p* = 0.074). The large effect sizes observed for these outcomes (*d* = 1.03 and 0.97, respectively) suggest meaningful effects that warrant replication in larger cohorts. Given the small sample, the findings of this study should be viewed as preliminary, and all conclusions should be interpreted with appropriate caution.
(2) The 4-week intervention duration, while sufficient to induce neural and metabolic adaptations, precludes any attribution of performance gains to structural muscle hypertrophy. Longer training periods would be needed to evaluate potential morphological changes. Additionally, direct physiological measurements (e.g., EMG, muscle biopsies) were not collected; therefore, the mechanistic interpretations offered herein remain inferential and should be verified in future research.
(3) The My Jump 2 application, although reliable for detecting within-subject changes, was not validated against a gold-standard linear position transducer for bench press power assessment. Consequently, the absolute MPP values reported in this study lack external validity and are not directly comparable to absolute power values obtained with validated instruments (e.g., linear position transducers, force plates). Absolute power values should therefore be interpreted as relative indicators of change. Furthermore, participants could not be blinded to group allocation due to the nature of the training intervention; however, standardized encouragement and objective outcome measures were employed to minimize motivational bias.
(4) This study included only elite male boxers, and the generalizability of these findings to female athletes, junior boxers, or athletes of different competitive levels remains to be established.
(5) The experimental group performed additional all-out punching bag training (RPE-10) while the control group continued with conventional technique training (RPE-5). Therefore, the observed improvements may be attributable to the increased overall training load rather than the specific nature of the RST stimulus. However, the control group also received additional technical training, and the specificity of improvements in anaerobic tasks (Wingate, 30 s punching) rather than single-effort peak velocity suggests that the RST stimulus, rather than merely increased volume, was responsible for the observed adaptations.

## Data Availability

The original contributions presented in the study are included in the article/Supplementary Material, further inquiries can be directed to the corresponding authors.
