# Correction: The effects of 4 weeks of sport-specific repeated sprint training on upper-body strength quality, anaerobic capacity and punching ability of elite male boxers

**DOI:** 10.3389/fspor.2026.1925581

**Published:** 2026-07-13

**Authors:** Tan Tian, Yongjie Niu, Shanjun Bao

**Affiliations:** 1 School of Sports Training, Wuhan Sport University, Wuhan, China; 2 School of Physical Education, Ningde Normal University, Ningde, China

**Keywords:** anaerobic capacity, boxers, high-intensity interval training, repeated sprint training, sport-specific, strength quality

There was a mistake in **Figure 1** as published. The published figure inadvertently contained a small watermark from an AI-powered image enhancement tool (Doubao AI), which was used solely for resolution improvement in response to the production team's request for higher-resolution figures. The image itself is a genuine photograph of the experimental equipment, and no scientific content was altered. The corrected **Figure 1** appears below.

**Figure 1:**
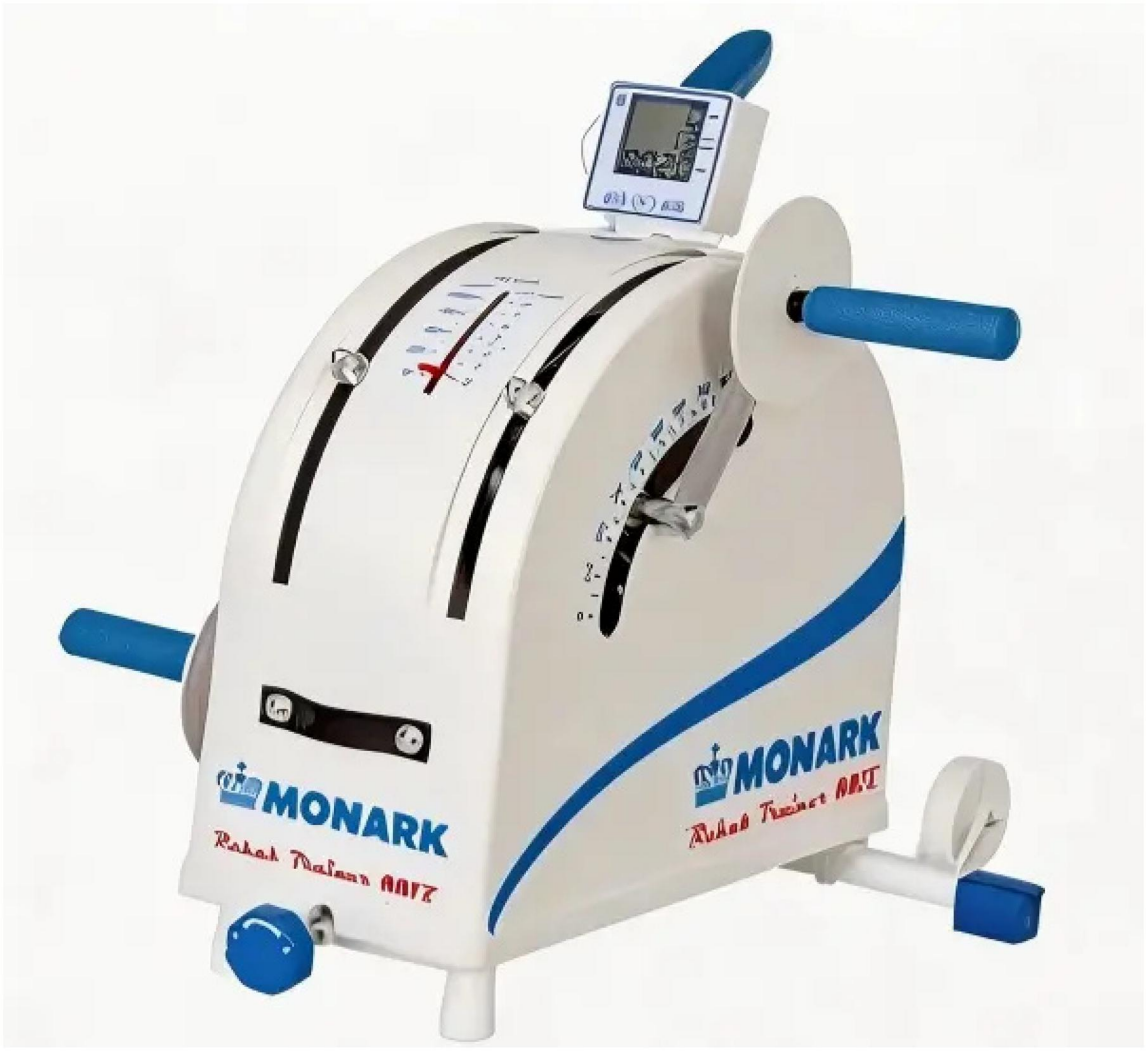
Monark894E upper-body wingate test cycle ergometer.

The **Generative AI statement** was erroneously given as:

“The author(s) declared that generative AI was not used in the creation of this manuscript.

Any alternative text (alt text) provided alongside figures in this article has been generated by Frontiers with the support of artificial intelligence and reasonable efforts have been made to ensure accuracy, including review by the authors wherever possible. If you identify any issues, please contact us.”

The correct **Generative AI statement** is:

“The author(s) declared that generative AI was used in the creation of this manuscript. The visual clarity of Figure 1 was enhanced using AI-assisted tools (Doubao AI) to meet publication standards. The scientific content of the image is unaltered, and the authors take full responsibility for its authenticity.

Any alternative text (alt text) provided alongside figures in this article has been generated by Frontiers with the support of artificial intelligence and reasonable efforts have been made to ensure accuracy, including review by the authors wherever possible. If you identify any issues, please contact us.”

The original version of this article has been updated.

